# The Awareness and Attitude of Parents towards the Legislation of Child Restraint in Two Cities of China

**DOI:** 10.3390/ijerph17072405

**Published:** 2020-04-01

**Authors:** Ye Jin, Xiao Deng, Pengpeng Ye, Ji Peng, Juanjuan Peng, Lin Lei, Yan Yu, Leilei Duan

**Affiliations:** 1 The National Center for Chronic and Noncommunicable Disease Control and Prevention, the Chinese Center for Disease Control and Prevention, Beijing 100050, China; 2 Department of Chronic and Non-Communicable Disease Control and Prevention, Shenzhen Center for Chronic Disease Control, Shenzhen 518020, China; 3 Shanghai Center for Disease Control and Prevention, Shanghai 200336, China

**Keywords:** child restraint, legislation, attitude

## Abstract

The death of child passengers was one of the leading causes of death among children fatally injured on roads in China. Child restraint can effectively protect child passengers. Mandatory child restraint law has been enacted locally in Shanghai and Shenzhen, two major cities in China. In order to understand the public attitude on national legislation in these cities, we conducted a cross-sectional survey with a sample of parents/caregivers with a child aged 0–6 years and own private car from Shanghai and Shenzhen. We used descriptive statistics to describe the distribution of parental awareness and attitudes towards the legislation of child restraint. There were less than 50% parents who were aware of the local legislation of child restraint use. Even though only around 20% of parents were able to respond accurately to the age standard in legislation, among those who knew of the legislation, most of the parents understood that the law had enforcement measures. More than 70% of parents supported the national legislation of child restraint use, and, among them, around 70% supported enforcement and punishment. Thus, the study provided supportive evidence for national legislation, but it also put forward that the work of popularizing law should be strengthened.

## 1. Introduction

Child injury has become a public health issue attracting widespread concern in the world [1]. In China, road traffic injuries are the second leading cause of death in children aged 1–14 years old [2]. Among those children who were killed in various modes of transport on the road, the majority were child pedestrian and child passengers [3]. Child restraint is one of the most important and useful measures that can prevent child occupants from injury and death in a crash. The use of child restraint can reduce the risk of death by at least 60% [4,5]. The use of child restraint is very usual in many countries, especially in high-income countries, while the utilization rate of child restraint in China has been relatively low [6,7,8]. It has been established that a mandatory child restraint law with enforcement is effective at promoting child restraint use [9]. For example, in 1978, Tennessee passed the law that children under the age of 4 must use child restraint. After legislation, the use rate of child restraint increased from 8% to 30%, and the child passenger mortality rate decreased by half [1]. Worldwide, there were 84 countries that have a national mandatory child restraint law [4]. Even though a national mandatory child restraint law has not been enacted yet in China, some provincial- or city-level regulations have been put in place [10,11,12]. This includes the cities of Shanghai and Shenzhen.

The purpose of this study was to explore the awareness and attitude of parents toward legislation in these two cities, Shanghai and Shenzhen, where mandatory child restraint regulation has been formulated and implemented.

## 2. Materials and Methods 

### 2.1. Mandatory Child Restraint Regulations in Shanghai and Shenzhen: 

“Regulations of Shanghai Municipality on Road Traffic Administration” was promulgated by Shanghai Municipal People’s Congress in 2016. Article 34 was “when driving a motor vehicle on the road, there shall be no following behaviors: when driving a family vehicle with a juvenile under the age of four, the child restraint is not equipped or not used correctly” [10].

“Regulations of Shenzhen Special Economic Zone on punishment of illegal acts of road traffic safety” was promulgated by Shenzhen Municipal People’s Congress in 2015. Article 11 was “when driving a motor vehicle with children, if children under the age of 12 ride in the copilot, or children under the age of four who take in small, micro non-operating passenger vehicles do not use child restraint that meets the national standards, shall be imposed a fine of 300 CNY (equal to 44 US dollars)“ [11].

### 2.2. Investigation Method: 

A cross-sectional survey, with a city-level representative sample of parents from Shanghai and Shenzhen cities, was conducted by using a tailored questionnaire to collect data surrounding the status of child restraint use and parental attitude toward the national mandatory child restraint law of 2018. The inclusion criteria were as follows:

- Should have at least one child aged 0–6 years in the family.
- Should have at least one private car in the family.
- Has driven the child aged 0–6 years out most often or accompanied the child in the private car most often.

The consent was obtained through reading an introduction “if you answer the questionnaire, you are considered to consent to taking the survey” in the front of questionnaire and answering the questionnaire.

The protocol of this study was approved by the Ethics Committee of the Center for Chronic and Noncommunicable Disease Control and Prevention, the Chinese Center for Disease Control and Prevention.

### 2.3. Investigation Content: 

The content of the questionnaire was compiled by the project team after literature review and expert consultation and was tested by pre-survey. It mainly includes the socio demographic characteristics, the status of children travelling by car, the use of child restraint, the awareness of local legislation on mandatory child restraint use, and the attitude towards the national law of mandatory child restraint use [9,10,11]. In this paper, we are only interested in the results of the awareness of local legislation and the attitude to the national law.

As for the awareness of local legislation, there were three choice questions. (1) ‘Are you aware of *** law with item about mandatory child restraint use?’; (2) ‘Do you know that the law has enforcement and punishment on mandatory child restraint use?’; and (3) ‘Do you know the age criteria of mandatory child restraint use in this law?’

As for the attitude to the national law, there were five questions. (1) ‘Do you support the national legislation on mandatory child restraint use?’ (choice question); (2) ‘Which do you prefer to be the criteria for the legislation?’ (choice question); (3) ‘What do you think should be use as an age/height criteria?’ (open-end question); (4) ‘Do you support for the enforcement and punishment on mandatory child restraint use in the national law?’ (choice question); and (5) ‘What types of enforcement and punishment do you think is appropriate? (Multiple choice question).

### 2.4. Sample Size: 

For each city, the sample size for different age groups (0–3 years and 4–6 years) was separately calculated since the sampling methods and procedures were different for those two age groups. The software PASS was used for sample size calculation. The parameter used in the sample size calculation for the survey is as follows: (1) α = 0.05; (2) Assuming that the always use rate of child restraint was lower than the rate of awareness of local legislation and the rate of support national law, the child restraint always used rate was applied in the sample size calculation, which was 30% and 12% for 0–3-year-old children in Shanghai and Shenzhen, respectively, and 20% and 7% for 4–6-year-old children in Shanghai and Shenzhen, respectively, based on the previous study results on the use of child restraint in these two cities of China in 2014 [6]; (3) The relative error estimated for Shenzhen sampling was 20%, while the one estimated for Shanghai was 13%. The relative error in Shanghai was estimated to be lower for reducing the estimation interval of the use rate in Shanghai, which was higher than that of Shenzhen; (4) The design effect was assumed to be 2; (5) The effective response rate was estimated as 90%. 

The sample size of 0–3-year-old Shanghai and Shenzhen was about 1179 and 1566, respectively, and that of 4–6-year-old Shanghai and Shenzhen was about 2021 and 2836, respectively. Therefore, the minimum sample size in Shanghai and Shenzhen was 3200 and 4402, respectively.

### 2.5. Sampling Methods and Procedures

For the parents with children aged 0–3, since they were required by the doctors to regularly take their child to the community health service center for vaccination, the sample was obtained through community health service center. For the parents with children aged 4–6, the sample was obtained from kindergartens.

A two-stage cluster random sampling was used. For children aged 0–3, a probability proportional to size (PPS) cluster random sampling was used to randomly select 20 streets/towns from Shanghai and Shenzhen, respectively. For each street/town, a sample of 70 parents with a 0–3-year-old child meeting the inclusion criteria and a sample of 90 was extracted according to ratio of age from vaccination clinics in a community health service center in Shanghai and Shenzhen, respectively. For children aged 4–6, a PPS cluster random sampling was used to select 20 kindergartens from Shanghai and Shenzhen, respectively. According to ratio of age, each kindergarten from Shanghai and Shenzhen extracted a sample of 145 and 160 parents, respectively. The parents must have a child aged 4–6 and meet the inclusion criteria.

### 2.6. Respondents Recruitment

For the parents of children aged 0–3 years, when they took their child to the community health service center for vaccination, they were asked if they had a 0–3-year-old child and owned a private car. The parents meeting criteria were invited to attend the survey and handed a paper questionnaire to answer. The parents answered the questionnaire when they were waiting for vaccination and gave it back to the community health service center.

For the parents of children aged 4–6, according to the information that the kindergarten provided in advance, the target respondents were randomly extracted from the parents with 4–6-year-old children and one car. The paper questionnaires were handed to the target parents by teachers when the parents went to the kindergarten to pick up their children, and the parents answered the questionnaire by themselves and gave it back to the teachers.

### 2.7. Analysis

The missing data and logic error were checked and confirmed with the respondents during the collection of the paper questionnaires by trained staffs from community health service centers and kindergartens. The responses were excluded from analysis if the error was not revised.

The sampling weight was calculated based on the two-stage cluster sampling method. A weighted percentage was used to describe the responses to the awareness of the local legislation and the support of national legislation. 

## 3. Results

### 3.1. Demographic Characteristic

There were 4201 response of parents from Shanghai and 4911 from Shenzhen included in the analysis (Table 1). The response rate was around 98% for each city. The distribution of characteristics was mostly similar in Shanghai and Shenzhen. The education levels of parents were mainly University (specialized subject) and University. The incomes of the families were principally >1442 and ≦2883 dollars/month. The gender ratio of the children was close to 1:1. The child age ratio of 0–3 to 4–6 was nearly 1.5:1. More children travelled by car 2–3 times a week. The rate of always using a child restraint in Shanghai was 40.79%, and 28.75% in Shenzhen (Table 1).

### 3.2. Awareness of Local Legislation in the Two Cities

According to the data, less than half of the parents (Shanghai 46.24%, Shenzhen 44.15%) knew the local legislation on the compulsory use of child restraint. In Shanghai and Shenzhen, 85.22% and 93.22% of the parents who were aware of legislation knew that the law had a punishment. Among the parents who were aware of the law, a little more than 20% (21.88% in Shanghai and 21.72% in Shenzhen) of parents knew that the age of using child restraint stipulated by the law was “under 4 years old”. In total, 53.35% in Shanghai and 41.02% in Shenzhen of parents thought it should be “under 12 years old” (Table 2).

### 3.3. Support of National Legislation in the Two Cities

More than 70% of parents in both cities (73.80% in Shanghai and 73.66% in Shenzhen) supported national legislation. About 60% of parents (58.42% in Shanghai and 61.76% in Shenzhen) thought that age should be used as the legislative standard for specifying whether child restraint should be used, and about 25% of parents (24.87% in Shanghai and 28.16% in Shenzhen) thought that height should be used as the legislative standard (Table 3).

**Figure 1:**
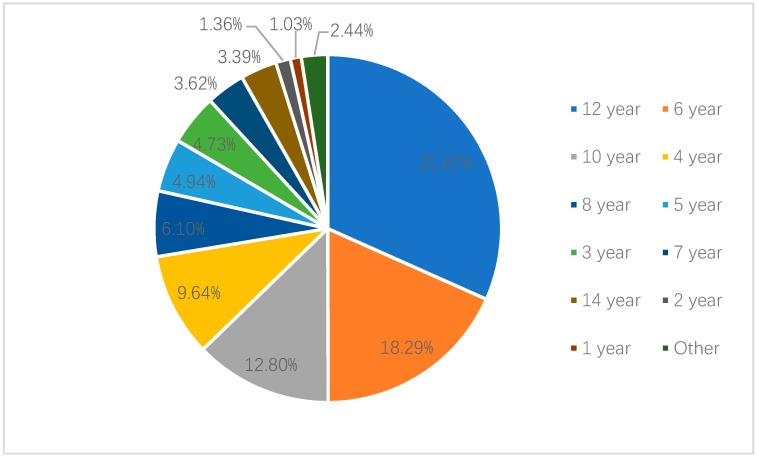
Recognized age criteria of national child restraint legislation by parents in Shanghai.

**Figure 2:**
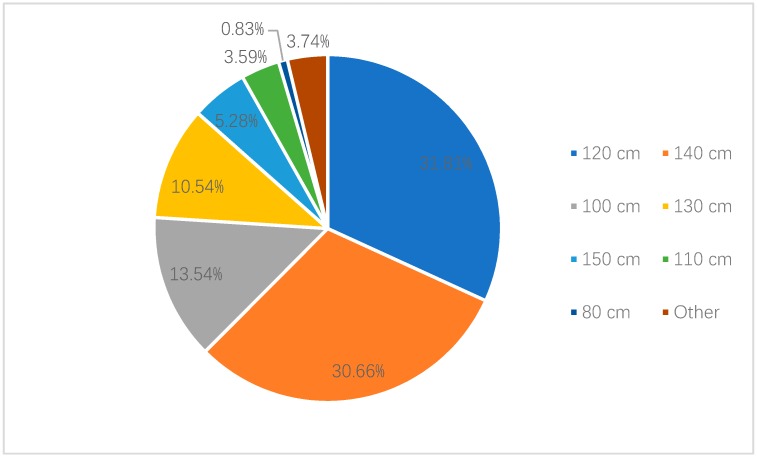
Recognized height criteria of national child restraint legislation by parents in Shanghai.

Among the parents who thought that they should use age as the standard, 31.67% of parents in Shanghai thought that the age standard should be “under 12 years old”, while 18.29% thought that it should be “under 6 years old”. In Shenzhen, 23.27% of parents who considered age as the standard believed that “under 12 years old” was the most appropriate age threshold. In total, 22.49% believed “under 6 years old” should be the age standard. Only 9.64% and 9.25% of the parents in Shanghai and Shenzhen, respectively, knew that the age criterion was “under 4 years old” (see Figure 1 and Figure 2).

**Figure 3:**
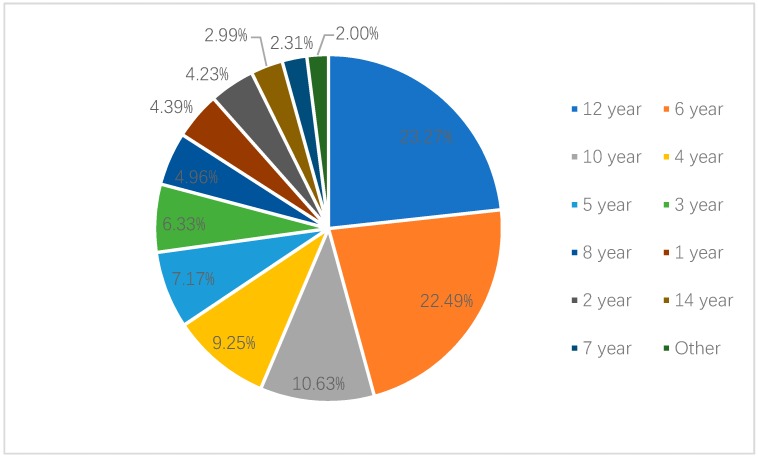
Recognized age criteria of national child restraint legislation by parents in Shenzhen.

**Figure 4:**
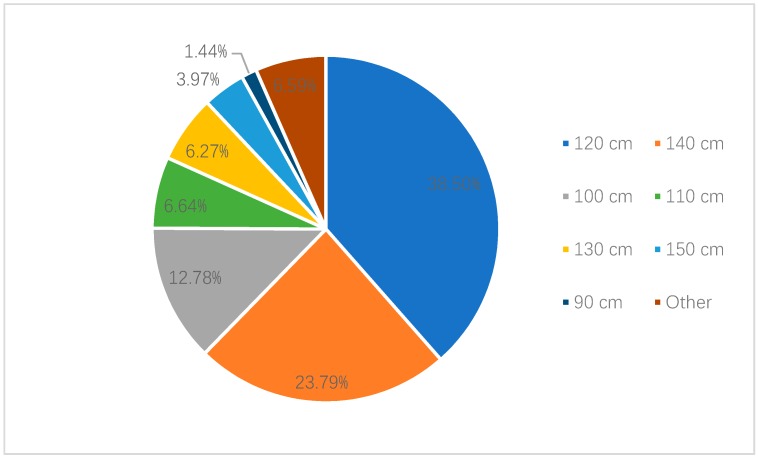
Recognized height criteria of national child restraint legislation by parents in Shenzhen.

Among the parents who believed height should be the legislative standard, 31.81% parents in Shanghai thought the height standard should be “below 120 cm”, while 30.66% thought it should be “below 140 cm”. A total of 13.54% and 10.54% parents thought it should be “below 100 cm” and “below 130 cm”, respectively. In Shenzhen, 38.50% parents thought that it should be “below 120 cm”, while 23.79% believed it should be “below 140 cm”. Moreover, 12.78% parents thought that it should be “below 100 cm” (Figure 3 and Figure 4).

Of the surveyed parents in Shanghai and Shenzhen, 73.62% and 78.64% supported the punishment of the behavior of not using the car seat, respectively. Among the parents who supported the punishment, about 45% in Shanghai and Shenzhen supported education (40.84% in Shanghai, 46.71% in Shenzhen), around 25% of the parents supported deduction on violation behavior (28.06 in Shanghai, 22.53% in Shenzhen), and 78% of parents supported a fine (78.06% in Shanghai, 78.42% in Shenzhen). Among the parents who supported the fine, a little more people in Shanghai supported the fine of more than 44 dollars (21.53%) and 15–28 dollars (21.45%), and a little more people in Shenzhen preferred the fine of more than 44 dollars (23.83%).

In addition, about 85% of the parents (84.44% in Shanghai and 86.08% in Shenzhen) thought that, if a child was not using a car seat in a crash that led to the injury of a child in the car, the parent or guardian of the child should take on a certain degree of responsibility for the crash. Among the parents who did not support the national legislation, about 45% (Shanghai 44.84%, Shenzhen 47.84%) thought that since it was their own business to use the child restraint, no legislation was needed.

## 4. Discussion

Shanghai and Shenzhen are two developed cities. Through the investigation in the two cities that took the lead in legislation of child restraint use, this paper aimed to show the public’s awareness and attitude to the law after the legislation was enacted in these two cities, and to provide evidence of parental views for the introduction of legislation in other cities, even for the national legislation.

### 4.1. Awareness of Legislation:

The results show that although the two cities have legislation in place, nearly 50% of parents still did not know the law, demonstrating the need for a greater awareness-raising effort. Among the parents who knew the law, most of them also knew that there was law enforcement punishment, but few of them knew the right age standard of the law, which might be because that enforcement punishment could attract more attention on legislation. The result demonstrates that the law publicity needs to pay more attention on the details of the law. When publicizing the law, we should emphasize the details, so as to improve the accuracy of parents’ understanding of the law, which could help to reduce the violation of laws related to mandatory child restraint use due to the inaccurate cognition of parents. It has also been suggested that the publicity of law enforcement punishment might help to improve the effect of law popularization, which may be because enforcement and penalties are easier to get people’s attention [13,14]. 

Among the parents who knew the law, nearly half of them thought that the age standard of using the child restraint stipulated by the law was “under 12 years old”. Although this is different from the real age standard of current local legislation, it is consistent with the suggested age of using the child restraint according to the professional authority [15], indicating a widespread understanding of the benefit of child restraint use for all children. Overall, the results indicate that although there were some deficiencies in the publicity of law popularization, there appears to be good knowledge about the use of child restraints generally, which would help to promote the extension of legal coverage age to older children.

### 4.2. Support Status of National Mandatory Child Restraint Law

Whether or not they knew about the local legislation of child restraint use, more than 70% of parents in the two cities supported the national legislation, which indicated that the legislation in cities with similar economic level and culture is likely to get support. In addition, most parents agreed that in a crash that leads to the injury of a child in the car, if the child did not use the child restraint, the parents or guardians of the child should take certain degree of responsibility for the child traffic injury. This suggests that most parents agree that the use of the child restraint is the responsibility of the parents. The support of legislation and the recognition of responsibility suggested that parents understood and agree with the role of child restraint in protecting children’s passenger safety. However, the use rate of child restraint was still low. A previous study showed that children’s and family members’ refusal to use child restraint might be one of the reasons preventing parents from using child restraint for their children [16]. National legislation on mandatory child restraint use will enhance the authority of the role of child restraint in protecting the safety of child passengers. Popularizing authoritative law can help parents strengthen their confidence to persuade their children and other family members (especially elders) to use child restraint [17]. In addition, education about the skills of promoting children to accept the child restraint can also help reduce the resistance of children refusing to use the seat. Among the parents who did not support the national legislation, some of them thought that it was their own business whether they use the child restraint or not, so it did not need legislation. Therefore, it was suggested that when popularizing law, an important strategy might be to increase understanding among parents about the role legislation can play in motivating people to use child restraint for all children.

As a child grows and exceeds a certain limit, a child could use the adult seat belt. At present, there are mainly two popular opinions about how to define the standard of legislation for the compulsory use of child restraint, which are age or height. From a professional point of view, the basis for determining whether children should use child restraint or can use adult seat belts is height. In order to enable a child to be tied to the right body parts by seat belts just like adults, only children with a height of 145 cm and above can use seat belts [9]. Some countries used height as the legislative standard for the use of child restraint [18,19,20], while some used age as the legislative standard [21,22]. Although the height of about 145 cm is nearly about 12 years old, due to the different physical development speed of different children, the height and weight difference of children of the same age may be larger, so it was recommended to take height as the defining standard [23]. In the survey of two cities, more parents supported age as the limited standard for the use of child restraint, which might be because the local legislation of child car seat in both cities was based on age. Some parents supported height as the standard, and some of them even supported 140 cm as height standard, which is closed to the recommended 145 cm. This demonstrates a certain level of public opinion basis for national legislation based on height but more publicity on the use of height as a marker for when children can move into adult seat belts might be needed.

Law enforcement and punishment are important means to promote the effective implementation of child restraint legislation [24]. After local legislation and the enforcement of the law, a number of parents supported the enforcement of national child restraint legislation. Among those who supported law enforcement and punishment, more parents supported the form of punishment, fines, and education rather than points deducted for traffic violations. While there was no clear preference for the amount of the fine, the majority of respondents suggested more than 44 dollars. In addition, it should be noted that fines are not an end in itself. The purpose of legislative and enforcement penalties is to promote the use of child restraint by the public. Therefore, although fine punishment can promote correct behavior by increasing the illegal cost to some extent, it still needs to improve public awareness and change attitudes and behavior habits by combining with education, which could lead to longer term use of child restraint.

Currently, in China, 19 cities have local-level legislations on mandatory child restraint use. The national legislation has also been attached more importance. The public opinion of national legislation on mandatory child restraint use will be a crucial point considered in the progress of legislation. The results in this paper show the public opinions on national legislations of mandatory child restraint use in two developed cities in China to provide evidence for national legislation. More surveys from other cities with different economic levels were required to provide more and stronger evidence on public support on national legislation. At the same time, we should also notice that despite the fact that local legislation was enacted, there were also deficiencies in the publicity and education of legislation, resulting in a low awareness rate of local legislation. The usage of child restraint was still relatively low. Therefore, the following research may need to pay attention to how to improve the publicity of the law and child restraint use after legislation, and strengthen law enforcement. The affordability of the child restraint should also be a concern of future legislation, as well as the accessibility of guides and services for correctly choosing, installing, and using the child restraint and for training the skills required to persuade children to use the child restraint.

### 4.3. Limitation

There are some limitations in this study. Firstly, given the self-reported data, recall bias and mistakes could occur in the process of data collection. The awareness and attitude could also be partially affected due to the possibility of the Hawthorne effect in the process of data collection. Secondly, given the representative sample for population with private cars, the finding should be cautiously extrapolated to the general population in two cities. Thirdly, given the very high social-economic settings of Shanghai and Shenzhen, the findings of this study should be cautiously used as a reference in other researches.

## 5. Conclusions

Generally, the results show that most parents with private cars in Shanghai and Shenzhen supported the national legislation of child restraint, which could provide supporting evidence for the national legislation. In addition, since parents have a poor awareness of legislation, the work of law publicity still needs to be strengthened. Furthermore, since the two regions are economically developed, it is necessary to understand the attitude of other regions with a lower economic level to legislation.

## Figures and Tables

**Table 1:** Demographic characteristics.

Characteristic		Shanghai		Shenzhen	
		Number	Percentage (95%CI)	Number	Percentage (95%CI)
Education of parents	Primary school	3	0.082 (0.021, 0.33)	32	0.79 (0.49, 1.26)
	Junior middle school	157	4.46 (2.78, 7.08)	406	9.45 (7, 12.66)
	High school / Secondary vocational / Technical School	437	11.69 (8.92, 15.19)	1067	23.97 (19.96, 28.5)
	University (Specialized subject)	996	23.34 (21.23, 25.6)	1311	27.67 (25.09, 30.42)
	University	2201	51.32 (46.44, 56.18)	1766	33.63 (28.57, 39.08)
	Postgraduate	402	9 (7.07, 11.38)	295	4.03 (2.94, 5.5)
	Others	5	0.1 (0.032, 0.31)	34	0.46 (0.28, 0.74)
	Total	4198	100	4879	100
Income of the family	≦721 U.S. dollars /month	186	4.49 (3.26, 6.14)	346	7.92 (6.28, 9.94)
	> 721 & ≦1442 U.S. dollars /month	740	20.46 (17.31, 24.03)	1086	25.89 (22.95, 29.07)
	>1442 and ≦2883 U.S. dollars/month	1489	35.59 (32.55, 38.74)	1510	33.95 (31.72, 36.26)
	>2883 and ≦14,417 U.S. dollars /month	1567	34.18 (29.99, 38.64)	1262	21.17 (17.83, 24.93)
	>14,417 U.S. dollars/month and upper	64	1.68 (1.19, 2.35)	359	5.63 (4.75, 6.67)
	Unknown	148	3.61 (2.67, 4.85)	348	5.44 (4.38, 6.74)
	Total	4194	100	4911	100
Car price	≦14,417 U.S. dollars/month	337	8.53 (6.53, 11.07)	514	12.42 (10.21, 15.02)
	>14,417 and≦24,508 U.S. dollars /month	1662	39.83 (36.23, 43.55)	1561	36.1 (32.27, 40.12)
	>24,508 and≦36,042 U.S. dollars /month	1080	25.37 (23.16, 27.71)	1060	21.73 (19.35, 24.32)
	>36,042 and ≦50,458 U.S. dollars /month	597	14.84 (12.94, 16.96)	664	11.89 (10.23, 13.77)
	>50,458	417	9.26 (7.81, 10.95)	639	10.01 (8.17, 12.21)
	Unknown	108	2.17 (1.64, 2.87)	473	7.85 (6.21, 9.87)
	Total	4201	100	4911	100
Age of children	0–3 y	1346	57.53 (52.23, 62.66)	1777	63.28 (54.82, 70.99)
	4–6 y	2855	42.47 (37.34, 47.77)	3134	36.72 (29.01, 45.18)
	Total	4201	100	4911	100
Gender of children	Male	2187	51.51 (49.89, 53.12)	2598	53.18 (51.32, 55.04)
	Female	2014	48.49 (46.88, 50.11)	2313	46.82 (44.96, 48.68)
	Total	4201	100	4911	100
Weight of children	–	18.10 ± 5.38 kg		17.02 ± 5.18 kg	
Height of children	–	105.59 ± 16.74 cm		102.74 ± 17.09 cm	
Trip frequency of children	Every day / Almost every day	1150	23.33 (19.82, 27.26)	980	16.88 (13.23, 21.29)
	2–3 times a week	1836	43.05 (39.6, 46.57)	1623	33.84 (31.22, 36.55)
	2–4 times a month	949	25.73 (23.66, 27.91)	1459	31.29 (27.64, 35.19)
	Once a month or less	266	7.89 (6.25, 9.91)	849	18 (14.82, 21.69)
	Total	4201	100	4911	100
Travel distance of children	Less than 3 km	1114	26.28 (21.75, 31.38)	1395	26.86 (22.99, 31.12)
	3–5 km	1423	34.86 (31.84, 38)	1404	28.98 (25.26, 33.01)
	6–10 km	986	23.27 (20.27, 26.57)	1184	25.27 (22.71, 28.01)
	10km and upper	677	15.59 (13.2, 18.31)	928	18.89 (16.04, 22.11)
	Total	4200	100	4911	100
Driver wearing safety belt	Always	3708	87.77 (84.45, 90.47)	4267	86.93 (83.91, 89.45)
	Often	229	6.17 (4.59, 8.25)	317	6.19 (4.93, 7.74)
	Sometimes	140	3.08 (1.95, 4.84)	172	3.68 (2.49, 5.4)
	Seldom	81	1.85 (1.32, 2.6)	121	2.33 (1.83, 2.96)
	Never	43	1.12 (0.69, 1.8)	34	0.87 (0.56, 1.37)
	Total	4201	100	4911	100
Utilization rate of child restraint	Always	1609	40.79 (35.94, 45.83)	1536	28.75 (24.73, 33.13)
	Often	497	12.53 (10.4, 15.03)	528	10.64 (9.25, 12.2)
	Sometimes	406	8.94 (7.42, 10.74)	508	9.78 (8.32, 11.47)
	Seldom	483	10.89 (9.43, 12.55)	546	11.08 (9.7, 12.63)
	Never	288	6.21 (4.97, 7.73)	238	5.15 (4.2, 6.3)
	Not own child restraint	918	20.64 (16.38, 25.66)	1555	34.61 (28.81, 40.91)
	Total	4201	100	4911	100

**Table 2:** Awareness of local legislation in the two cities.

Variables	Shanghai		Shenzhen	
	Number	Percentage (95%CI)	Number	Percentage (95%CI)
**Awareness of legislation**				
Unknown	2015	53.76 (49.17, 58.29)	2535	55.85 (49.99, 61.55)
Known	2186	46.24 (41.71, 50.83)	2373	44.15 (38.45, 50.01)
Total	4201	100	4908	100
**Awareness of enforcement and punishment**				
Unknown	328	14.78 (12.41, 17.52)	123	6.78 (4.67, 9.74)
Known	1858	85.22 (82.48, 87.59)	2235	93.22 (90.26, 95.33)
Total	2186	100	2358	100
**Awareness of age criteria of legislation**				
Under 2 years	53	2.29 (1.54, 3.41)	58	3.53 (2.42, 5.12)
Under 4 years	516	21.88 (19.1, 24.93)	600	21.72 (19.16, 24.51)
Under 6 years	230	10.13 (8.59, 11.92)	371	15.61 (12.11, 19.89)
Under 8 years	53	2.6 (1.83, 3.67)	54	2.4 (1.76, 3.27)
Under 10 years	47	2.12 (1.42, 3.13)	44	2.37 (1.65, 3.39)
Under 12 years	1131	53.35 (49.43, 57.24)	947	41.02 (38.19, 43.92)
Other	6	0.19 (0.064, 0.55)	13	0.48 (0.23, 0.99)
Unknown	150	7.44 (5.64, 9.76)	276	12.87 (10.74, 15.34)
Total	2186	100	2363	100

**Table 3:** Support of national legislation in the two cities.

Variables	Shanghai		Shenzhen	
	Number	Percentage (95%CI)	Number	Percentage (95%CI)
**Support for national legislation**				
Yes	3113	73.8 (70.73, 76.66)	3549	73.66 (71.08, 76.1)
No	417	9.29 (7.88, 10.92)	625	11.79 (10.43, 13.29)
Indifferent	671	16.91 (14.69, 19.4)	737	14.55 (12.75, 16.55)
Total	4201	100	4911	100
**Criteria for legislation**				
Age	2065	58.42 (52, 64.57)	2864	61.76 (59.2, 64.26)
Height	1349	24.87 (20.28, 30.11)	1473	28.16 (25.49, 30.99)
Other	75	1.33 (0.88, 2)	51	1.11 (0.68, 1.81)
Indifferent	544	15.38 (12.01, 19.48)	466	8.97 (7.64, 10.5)
Total	4033	100	4854	100
**Support for enforcement and punishment**				
Yes	2813	73.62 (71.11, 75.98)	3,360	78.64 (75.89, 81.15)
No	330	8.56 (7.13, 10.24)	365	8.23 (7.01, 9.64)
Indifferent	641	17.82 (15.47, 20.45)	555	13.13 (11.33, 15.18)
Total	4201	100	4905	100
**Types of enforcement and punishment**				
Fine	2215/2813	78.06 (73.34, 82.16)	2674/3360	78.42 (74.22, 82.1)
44 U.S. dollars and upper	563/2813	21.53 (18.62, 24.76)	824/3360	23.83 (20.78, 27.19)
29-43 U.S. dollars	462/2813	17.52 (15.6, 19.62)	676/3360	20.76 (17.08, 24.99)
15-28 U.S. dollars	666/2813	21.45 (18.6, 24.61)	704/3360	20.62 (16.96, 24.84)
Under 14 U.S. dollars	524/2813	17.56 (15.2, 20.19)	470/3360	13.2 (11.77, 14.78)
Deduction	823/2813	28.06 (24.95, 31.4)	790/3360	22.53 (17.64, 28.31)
Education	1171/2813	40.84 (36.18, 45.67)	1532/3360	46.71 (43.03, 50.44)
Other	15/2813	0.48 (0.26, 0.89)	34/3360	0.68 (0.41, 1.11)
